# Understanding Determinants of Consumer Mobile Health Usage Intentions, Assimilation, and Channel Preferences

**DOI:** 10.2196/jmir.2635

**Published:** 2013-08-02

**Authors:** Arun Rai, Liwei Chen, Jessica Pye, Aaron Baird

**Affiliations:** ^1^Center for Process Innovation and Department of Computer Information SystemsJ Mack Robinson College of BusinessGeorgia State UniversityAtlanta, GAUnited States; ^2^Center for Process InnovationJ Mack Robinson College of BusinessGeorgia State UniversityAtlanta, GAUnited States; ^3^Center for Health Information Technology and Institute of Health AdministrationJ Mack Robinson College of BusinessGeorgia State UniversityAtlanta, GAUnited States

**Keywords:** mobile health, consumer preferences, adoption, health information technology, multivariate analyses

## Abstract

**Background:**

Consumer use of mobile devices as health service delivery aids (mHealth) is growing, especially as smartphones become ubiquitous. However, questions remain as to how consumer traits, health perceptions, situational characteristics, and demographics may affect consumer mHealth usage intentions, assimilation, and channel preferences.

**Objective:**

We examine how consumers’ personal innovativeness toward mobile services (PIMS), perceived health conditions, health care availability, health care utilization, demographics, and socioeconomic status affect their (1) mHealth usage intentions and extent of mHealth assimilation, and (2) preference for mHealth as a complement or substitute for in-person doctor visits.

**Methods:**

Leveraging constructs from research in technology acceptance, technology assimilation, consumer behavior, and health informatics, we developed a cross-sectional online survey to study determinants of consumers’ mHealth usage intentions, assimilation, and channel preferences. Data were collected from 1132 nationally representative US consumers and analyzed by using moderated multivariate regressions and ANOVA.

**Results:**

The results indicate that (1) 430 of 1132 consumers in our sample (37.99%) have started using mHealth, (2) a larger quantity of consumers are favorable to using mHealth as a complement to in-person doctor visits (758/1132, 66.96%) than as a substitute (532/1132, 47.00%), and (3) consumers’ PIMS and perceived health conditions have significant positive direct influences on mHealth usage intentions, assimilation, and channel preferences, and significant positive interactive influences on assimilation and channel preferences. The independent variables within the moderated regressions collectively explained 59.70% variance in mHealth usage intentions, 60.41% in mHealth assimilation, 34.29% in preference for complementary use of mHealth, and 45.30% in preference for substitutive use of mHealth. In a follow-up ANOVA examination, we found that those who were more favorable toward using mHealth as a substitute for in-person doctor visits than as a complement indicated stronger intentions to use mHealth (*F*_1,702_=20.14, *P*<.001) and stronger assimilation of mHealth (*F*_1,702_=41.866, *P*<.001).

**Conclusions:**

Multiple predictors are shown to have significant associations with mHealth usage intentions, assimilation, and channel preferences. We suggest that future initiatives to promote mHealth should shift targeting of consumers from coarse demographics to nuanced considerations of individual dispositions toward mobile service innovations, complementary or substitutive channel use preferences, perceived health conditions, health services availability and utilization, demographics, and socioeconomic characteristics.

## Introduction

### Background

Mobile health (mHealth) is defined as, “using wireless mobile communication technology to aid health services delivery” [1]. According to a recent health care market research study, 31% of US adults have used their mobile phones for accessing health information [2]. In addition, 19% of US adults who own a smartphone have at least 1 health application on their phone, with exercise, diet, and weight apps among the most popular [2]. Approximately half of the patients surveyed in a recent mHealth opinion survey believed that mHealth could increase their control over their health care, provide more convenient access to needed health information, and ultimately improve their health care costs and quality [3]. Such results are not surprising because mHealth can provide many benefits, including portable access to continuous streams of information and powerful interactive functionality driven by devices that often support a wide array of health applications [4]. However, questions remain as to what determines whether consumers will use and assimilate mHealth and whether or not channel preferences will play a significant role.

The introduction of mHealth represents a drastic shift in focus from traditional medical informatics based on industrial age concepts (eg, provider driven) to consumer health informatics based on the ubiquity of information and interconnected mobile computing infrastructure [5]. In practice, mHealth is often used for transmitting electronic medical records between medical staff and patients [6], monitoring patients remotely [6,7], sending electronic alerts for disease control [8], and providing useful applications, information, and functionality to health consumers [2]. The general category of mHealth innovations considered in this paper are typically used by consumers for activities relating to obtaining health advice (eg, the WebMD mobile app [9]), promoting compliance and adherence to medical treatments (eg, the iPharmacy Pill ID & Rx Reminder app [10]), staying connected with health care provider(s) (eg, the Mayo Clinic Patient app [11] and the eClinicalMobile app [12]), personal health management (eg, the GoMeals app [13], the Livestrong app [14], and the WellDoc app [15]), and chronic disease management (eg, the Glucose Buddy app [16] for diabetics).

Research in the mHealth context has demonstrated that intrinsic motivations facilitate mHealth adoption whereas perceived risks, such as perceived privacy risks and perceived psychological risks associated with making choices that may be regretted later, can inhibit mHealth adoption [17]. Perceptions and attitudes toward mHealth have been shown to positively affect an individual’s intention to use these types of services [18]. It has been suggested that the digital revolution brought by mobile and other technology has enriched doctor-patient communications [19]. Use of gamification in mHealth has recently been shown to increase glucose monitoring in diabetic adolescents [20]. Studies have also examined mHealth trends and associated risks [21,22], the impact of mHealth interventions on outcomes in specific clinical areas (eg, smoking cessation [4], HIV [23], and diabetes [24]), economic implications of mHealth usage (eg, [25]), and the use of mHealth to broaden access to health care in developing countries (eg, [26,27]).

Although expectations of the transformative (and disruptive) potential of mHealth are enormous and research is expanding in this area, little is known about how this digital health care service channel is viewed by consumers, given a traditionally hands-on provider-patient direct service channel. Given recent calls for more consumer health informatics research, especially in regards to consumer information seeking needs and behaviors [28], mHealth [29], and our presently limited knowledge of how consumers’ traits and health perceptions affect consumers’ mHealth usage intentions, assimilation, and channel preferences, our study is motivated by the substantial research opportunities in this interesting and emerging space. We specifically focus on what determinants are associated with consumer mHealth usage intentions, assimilation, and channel preferences.

### Theoretical Foundation and Research Model

Past work on the individual adoption of information technology (IT) has identified that consumer characteristics (eg, socioeconomic characteristics [30]), individual differences (eg, personal innovativeness [31,32]), and situational factors (eg, access to and utilization of health care services [33,34]) significantly impact IT adoption preferences. A recent systematic review of consumer health technology acceptance studies pointed out that many studies have assessed the effects of consumer traits (eg, age, income, education) on health technology acceptance, but theoretically motivated constructs, interaction effects (moderators), and health status variables have not yet been fully considered in consumer health technology acceptance studies [33]. Additionally, mHealth studies have not yet jointly examined consumer traits, health perceptions, and consumer preferences for mHealth as a substitute or complement to in-person doctor office visits.

**Figure 1:**
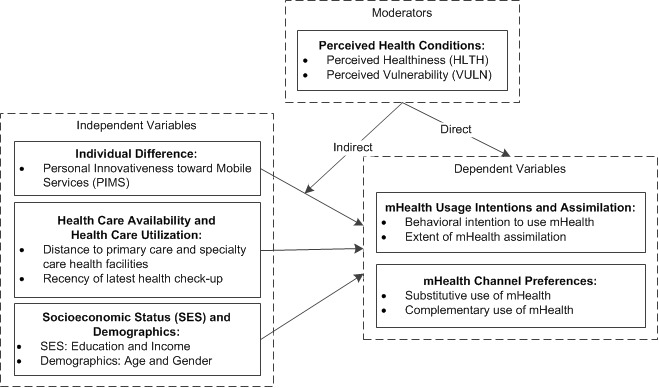
Research model.

Drawing upon technology adoption [33,35], technology assimilation [36,37], consumer behavior [38,39], and health informatics literature (eg [33,40-42]), we seek to fill this gap by focusing on determinants of consumer mHealth usage intentions, assimilation, and channel preferences in the United States. We aim to contribute to the health informatics and mHealth literature by assessing the following: (1) predictors of consumer mHealth usage intentions and assimilation, including personal innovativeness toward mobile services (PIMS), health care availability, health care utilization, socioeconomic status, and demographics, (2) consumer preferences for mHealth as a substitute or complement to in-person provider-patient interactions, and (3) the direct and interactive (moderating) effects of perceived health conditions (Figure 1).

### Dependent Variables: Behavioral Intention to Use mHealth and Extent of mHealth Assimilation

Based on the literature on technology acceptance [33,35] and technology assimilation [36,37], we conceptualize 2 dependent variables associated with consumers’ mHealth acceptance: (1) mHealth usage intentions (ie, intention to use mHealth for nonadopters and intention to continue using mHealth for adopters) and, (2) mHealth assimilation (ie, combined, staged measure ranging from extent of awareness to frequency of use). Self-reported behavioral intention to use an information system is a widely used dependent variable in technology acceptance research (eg, [43]) and is designed to measure intention of initial technology usage or continued usage (referred to collectively as *intention to use* for the remainder of this paper). Technology assimilation is often used as an indicator of the process of learning and applying a technology through multiple stages, ranging from very early stages of evaluation of options (awareness) to later stages of extensive use in which the technology has been incorporated into routines (frequent use) [44] (referred to collectively as *assimilation* for the remainder of this paper). The use of these 2 dependent variables in our analyses is meant to explore commonalities and differences in predictors between self-reported mHealth usage intentions and self-reported mHealth assimilation.

### Dependent Variables: mHealth Substitutive and Complementary Use

Drawing from consumer behavior literature [38,39], we consider 2 dependent variables that reflect consumers’ mHealth channel preferences: (1) substitutive use (ie, the willingness to use mHealth as a replacement for in-person doctor visits) and, (2) complementary use of mHealth (ie, the willingness to use mHealth to augment in-person doctor visits). Consumer behavior literature has suggested that offering alternative channels for service consumption (eg, self-service technologies) affords many conveniences and benefits to consumers [45,46]. However, such conveniences and benefits may come at a cost of taking the time to learn how to use the new channel, expending additional individual effort perhaps not required when interacting in-person, and concerns about overall service performance given the new production and consumption medium [47]. Given that health care has traditionally been conducted via hands-on in-person interactions, an important consideration is whether or not consumers will accept technology intermediation. Therefore, we assess consumers’ willingness to use mHealth as either a substitute or a complement to in-person doctor visits, irrespective of whether or not they are currently mHealth users.

### Independent Variable: Personal Innovativeness Toward Mobile Services

We draw on marketing and information systems research to identify personal innovativeness toward mobile services (PIMS) as a key individual difference for evaluating consumer adoption of technology innovations. Based on prior work [31,48-50], we define PIMS as the degree to which an individual is willing to try out any new mobile technology service. We suggest that PIMS is a proxy for personal innovativeness toward information technology (PIIT) [31] in the context of mobile services. Prior research on PIIT has suggested that individuals who are more open to experiences with technology typically have stronger technical self-efficacy (positive beliefs and abilities associated with technical tasks, such as using a computer) [51,52]. Those with higher PIIT are often associated with higher levels technology use, such as Internet and e-commerce use [53-55]. Recent studies have found personal innovativeness to be also a positive predictor in models assessing acceptance of mobile services [56,57]. Given that the delivery of health services on mobile platforms is currently at the very early stages of diffusion, it is likely that individuals with a predisposition for seeking out the latest innovations are more likely to initially adopt and use mHealth. Therefore, we extend the examination of PIIT to the context of mHealth using the PIMS construct.

### Independent Variables: Perceived Health Conditions

Health promotion and prevention research has widely acknowledged that individual beliefs about health conditions (eg, perceived healthiness and perceived vulnerability to chronic disease) predict individuals’ health behaviors (see reviews by [40,42]). We expect that consumers who feel healthier may be more open to trying health innovations. We base this expectation on evidence that those with lower perceived health may already have strong preexisting relationships with service providers (physicians and clinicians, in our case) and established therapeutic routines that may result in resistance to alternative service delivery and consumption options [46]. We also expect that people who feel more vulnerable to chronic diseases (eg, diabetes, heart disease, cancer, high blood pressure, and stroke) will have stronger needs and motivations to use health innovations. This expectation is based on evidence suggesting that those who currently report using mHealth often do so to mitigate negative long-term health consequences associated with health risk factors such as high blood pressure, obesity, inactivity, and high blood glucose levels [2].

### Moderating Effects: Personal Innovativeness Toward Mobile Services and Perceived Health Conditions

Prior research on technology acceptance has shown that PIIT and compatibility with work style (CMP) typically have positive interactive (PIIT*CMP) effects on technology usage intentions, suggesting that higher PIIT combined with higher CMP has an even greater positive impact on technology usage intentions than PIIT alone (eg, [48]). Compatibility is typically viewed as the degree of congruence between the innovation and the adopter’s preferences, needs, past experience, and/or values (eg, [58,59]). Drawing upon this literature, we propose the perceived health condition of an individual as a proxy for CMP, given that perceived health conditions often reflect health care needs (eg, [60]). We expect PIMS and health conditions to have both direct and indirect (moderating) effects. Specifically, we propose that PIMS and perceived healthiness (PIMS*HLTH) and PIMS and perceived vulnerability to chronic disease (PIMS*VULN) will have significant interactive effects on mHealth usage intentions, assimilation, and channel preferences.

### Independent Variables: Health Care Availability and Health Care Utilization

Consumers’ situational characteristics have been found to affect their technology adoption preferences [33] and product attraction and avoidance [61]. Specifically, access to health care and health care utilization have been considered in prior studies as important predictors and controls of technology adoption in the consumer health context (eg, [41]). However, the direction of the influence of these situational factors on consumers’ disposition to health care technologies and technology services channels, such as mHealth, is unclear. Frequent usage of in-person health services may imply that strong relationships have been established with health providers and technology intermediation may only be considered when patients are dissatisfied with their providers (eg, [62]). However, a recent study suggested the opposite and those who had strong relationships with their providers were more likely to use personal health records [63]. Therefore, we consider variation in the following consumer-level situational characteristics specific to health care availability and utilization: (1) distance to health facilities (both primary care and specialty facilities) as a proxy for health care access and, (2) whether or not the last health checkup was recent as a proxy for recent routine health care utilization.

### Independent Variables: Socioeconomic Status and Demographics

Although research results on the influence of socioeconomic status (SES) and demographic variables on innovation adoption are sometimes mixed (eg, [33]), in general, younger people [64], people with higher levels of education [65,66], and people with higher levels of income [65] are often found to be more innovative toward technology, including mobile services. However, mHealth also has the potential to be attractive to those who do not have a computer and Internet connection at home, but are still users of convenient mobile services (eg, [67,68]). Therefore, we account for the influence of SES and demographic characteristics on mHealth usage intentions, assimilation, and channel preferences by considering age, gender, income, and education in our research model.

## Methods

### Survey Design, Development, and Pretesting

Based on our research model, we designed a cross-sectional survey to measure consumers’ mHealth usage intentions, mHealth assimilation, and preference to use mHealth as a complement or substitute to in-person doctor visits. In addition, data regarding PIMS, health care availability, health care utilization, health perceptions, SES, and demographics were collected. Existing instruments were applied whenever possible. All questions were adapted to the mHealth context. Extensive pretesting was conducted before final administration of the survey. We invited a total of 20 reviewers, including physicians, technologists, researchers, and managers working in or very familiar with the mHealth industry, to examine the survey instrument in detail before pilot-testing the survey. Although most of the expert feedback indicated that the questions were clear and easy to understand, necessary revisions were made according to their suggestions. After initial survey refinement, we conducted an online pilot study with 134 consumers in the United States to further assess the psychometric properties of the measures. Further refinements were made to the survey wording on the basis of pilot study results. A summary of final survey items and measures is available in Multimedia Appendix 1.

### Survey Sample, Recruitment, and Administration

To facilitate the data collection and administration process, we recruited a market research company. We worked closely with this company to ensure that the sample was stratified to represent the US population in terms of age, gender, education, and income (following the US census) and that nonresponse bias was minimized. Using the online panel from the market research company, 8673 invitation emails were sent in 5 successive waves during a 2-week data collection period. In an effort to obtain a final sample that was nationally representative, we systematically monitored the demographics of incoming responses in each of the 5 waves and compared the means of the aggregate demographics to US census distributions. Oversampling was conducted in the subsequent waves for underrepresented strata (including those in younger and less-educated strata) to yield a final sample that was reasonably representative of the US census.

Each participant was provided with a unique passcode to access the online questionnaire. This design protected personal information from unauthorized access and also prevented duplicate responses from the same individual. Reminder emails were sent to participants to encourage them to complete the survey within the fieldwork period. The potential for nonresponse bias was mitigated by placing an emphasis on obtaining a nationally representative sample, following up with nonresponders and requesting participation, and including key demographic (age and gender) and socioeconomic (education and income) variables in the final models. Institutional Review Board (IRB) approval was obtained before survey administration. All participants acknowledged informed consent before taking the survey. Each participant took approximately 20 minutes to complete all 34 questions on the 17 screens of the online survey.

### Analysis

Measurement quality of the multi-item measures was assessed through confirmatory factor analysis (CFA) and detailed assessment of construct reliability and validity. The details of the measurement quality analysis are available in the next section and further detail is available in Multimedia Appendix 2. Final estimation for the primary models was completed using hierarchical ordinary least squares (OLS) regressions with robust standard error estimation. Several additional models and tests (eg, 2-stage least square analyses, mediated models with the demographic variables affecting the dependent variables through PIMS as well as directly, models with additional interactions) were evaluated to examine the robustness of the OLS results. The results stood up to the robustness checks. The OLS estimations are reported for the primary results because of their straightforward interpretation. Secondarily, we used ANOVA to assess differences in mHealth usage intentions and mHealth assimilation between respondents who preferred substitutive use of mHealth more strongly than complementary use versus those who preferred complementary use of mHealth more strongly than substitutive use.

## Results

### Descriptive Statistics

Complete data from 1132 respondents were collected. We carefully examined the distribution of respondents in our sample and found it to be nationally representative as compared with the distributions reported in the 2012 US Census [69] (Table 1). We obtained a final response rate of 13.05%, which is similar to response rates obtained in other online surveys conducted in comparable contexts (eg, [41,63]). Early- and late-stage respondents differed only by age and education. These differences were expected because of later-stage purposive oversampling of underrepresented strata. As such, the early- versus late-stage respondent analyses did not reveal any evidence of nonresponse bias. We conducted a marker variable analysis [70] and did not find evidence of common method bias.

The sample was relatively balanced in terms of gender (513 male and 619 female). The average age was 45 years (range 18-86, SD 16.20); 227 respondents (20.05%) were older than 60 years. Most respondents lived more than 6 miles from general and specialized health care facilities. Respondents had varying levels of education and individual income, representing reasonable variation in socioeconomic status. Additionally, 611 of 1132 respondents (53.98%) felt that they were healthy or very healthy, and there was substantial variance among respondents on the level of concern for vulnerability to chronic disease. Of the 1132 respondents, 430 (37.99%) reported that they had started using mHealth and 215 individuals (18.99%) reported use of mHealth on a regular basis. Further, 532 of 1132 respondents (47.00%) indicated that they would use mHealth as a substitute to in-person doctor visits, whereas 758 individuals (66.96%) indicated that they would use mHealth as a complement.

**Table 1:** Sample characteristics (N=1132).

Variables and categories			Sample, n (%)	US Census (%)
**Demographics**				
	**Age (years)**			
		18-29	218 (19.27)	22.1
		30-39	269 (23.76)	17.1
		40-49	169 (14.93)	18.6
		50-59	249 (22.00)	17.9
		60-69	155 (13.69)	11.8
		≥70	72 (6.36)	12.5
	**Gender**			
		Male	513 (45.31)	49.2
		Female	619 (54.68)	50.8
**Socioeconomic status**				
	**Education**			
		Not a high school graduate	18 (1.59)	12.9
		High school graduate	211 (18.64)	31.2
		Some college, but no degree	344 (30.39)	16.8
		Associate’s degree	154 (13.60)	9.1
		Bachelor’s degree	286 (25.27)	19.4
		Advanced degree	119 (10.51)	1.5
	**Individual income (US $)**			
		Less than 24,999	430 (37.99)	55.0
		25,000-49,999	344 (30.39)	24.0
		50,000-74,999	214 (18.90)	22.0
		75,000-99,999	85 (7.50)	5.0
		≥100,000	59 (5.21)	5.0
**Health care availability**				
	**Distance to primary health care facility**			
		<1 mile	34 (3.00)	—
		1-5 miles	90 (7.95)	—
		6-10 miles	472 (41.70)	—
		≥11 miles	375 (33.13)	—
		Do not know	161 (14.22)	—
	**Distance to specialized health care facility**			
		<1 mile	86 (7.60)	—
		1-5 miles	57 (5.04)	—
		6-10 miles	341 (30.12)	—
		≥11 miles	381 (33.66)	—
		Do not know	267 (23.59)	—
**Health care utilization**				
	**Recent health checkup**			
		No	125 (11.04)	—
		Yes, with the past 5years	37 (3.27)	—
		Yes, within the past 3 years	128 (11.31)	—
		Yes, within the past 1 year	842 (74.38)	—

### Measurement Quality

Before conducting hierarchical multivariate OLS regression analyses of the response data, we performed a series of checks to ensure the quality of the survey measures. Multimedia Appendix 2 provides a summary of means, standard deviations, and correlations for all variables as well as reliability and validity measures for multi-item constructs (eg, composite reliabilities and average variances extracted). The CFA was performed using AMOS 7.0 to assess the measurement properties of the 4 multi-item constructs (behavioral usage intention, substitutive use, complementary use, PIMS) at the model and item levels [71]. The 4-factor model yielded an adequate model fit (comparative fit index=0.98, goodness-of-fit index=0.96, and standardized root mean square residual=0.03) [72]. The factor loadings for each indicator on its corresponding construct were greater than 0.70 and were significant at *P*<.05, thus supporting convergent validity. For each construct, the average variance extracted (AVE) was greater than 0.5, suggesting that the explained variance was more than the unexplained variance [73]. Additionally, the square root of the AVE for each construct was more than all its interconstruct correlations, thereby establishing discriminant validity [74]. In terms of reliability, Cronbach alphas and composite reliabilities were all greater than the recommended 0.70 level [75]. These results suggest that the measurement scales exhibit good psychometric properties.

### Data Analysis

Multivariate OLS regressions were used to analyze the determinants of mHealth usage intentions, assimilation, and channel preferences. We evaluated 4 models per dependent variable in hierarchical fashion: (1) demographic and SES variables, (2) model 1 plus health variables (ie, distance to primary and specialized health care facilities, perceived healthiness, perceived vulnerability, and recency of health checkup), (3) model 2 plus PIMS, and (4) model 3 plus interaction effects. We also controlled for whether or not the respondent currently used mHealth (adopter or nonadopter dummy variable) to obtain generalizable results across the pooled sample of adopters and nonadopters.

### mHealth Behavioral Usage Intention and Assimilation Results

Table 2 (models A1-A4) reports behavioral usage intention regression results and Table 3 (models B1-B4) reports assimilation regression results.

In models A1 and B1 (demographic and SES variables only), 40.31% and 23.04% of variation in behavioral usage intention and assimilation was explained, respectively. Older respondents were associated with a lower level of behavioral usage intention (beta=–0.02, *P*<.001) and a lower level of assimilation (beta=–0.04, *P*<.001). In addition, individual income had a significant positive association with both behavioral usage intention (beta=0.12, *P*=.009) and assimilation (beta=0.71, *P*<.001). Moreover, level of education was negatively associated with assimilation (beta=–0.11, *P*=.02), but not significantly associated with behavioral usage intention (beta=0.00, *P*=.97). When controlling for differences between mHealth adopters and nonadopters with a dummy variable (adopter=1, nonadopter=0) in the behavioral usage intention models (A1-A4), we found the adopter group to have significantly increased intentions to continue using mHealth as compared to nonadopters’ intentions to begin using mHealth.

In models A2 and B2, health care access, health care utilization, and perceived health condition variables were added to the models, resulting in 44.06% and 44.86% variance explained, respectively. Respondents who felt healthier were positively associated with behavioral usage intention (beta=0.30, *P*<.001) and assimilation (beta=0.71, *P*<.001). Respondents who felt more vulnerable to chronic disease were associated with stronger behavioral usage intention (beta=0.36, *P*<.001) and stronger assimilation (beta=0.90, *P*<.001). In addition, the recency of health checkup significantly increased the level of assimilation (beta=0.17, *P*=.001), but was not significantly associated with behavioral usage intention (beta=0.04, *P*=.90). Distance to primary and specialized facilities were not significant predictors of either behavioral usage intention (primary: beta=–0.05, *P*=.43; specialized: beta=0.09, *P*=.08) or assimilation (primary: beta=0.09, *P*=.14; specialized: beta=0.01, *P*=.82).

In models A3 and B3, PIMS was added, increasing the explained variance (∆*R* ^*2*^) for behavioral usage intention by 15.55% and for assimilation by 8.47%. The significant positive coefficients indicate that PIMS was positively related to both behavioral usage intention (beta=1.11, *P*<.001) and assimilation (beta=0.84, *P*<.001).

In models A4 and B4, the interaction between PIMS and perceived healthiness (ie, PIMS*HLTH) and the interaction between PIMS and perceived vulnerability (ie, PIMS*VULN) were added, increasing the explained variance (∆*R* ^*2*^) for behavioral usage intention by 0.09% and for assimilation by 7.08%. Although the main effects were significant (HLTH, VULN, and PIMS) predictors of both behavioral usage intention and assimilation, the interactions were not significant predictors of behavioral usage intention (PIMS*HLTH: beta=0.03, *P*=.41; PIMS*VULN: beta=0.06, *P*=.10), but were significant predictors of assimilation (PIMS*HLTH: beta=0.50, *P*<.001; PIMS*VULN: beta=0.43, *P*<.001). These results indicate that the main effects of PIMS, HLTH, and VULN are important factors that predict mHealth behavioral usage intention. Additionally, these factors not only independently, but also jointly, influence assimilation.

**Figure 2:**
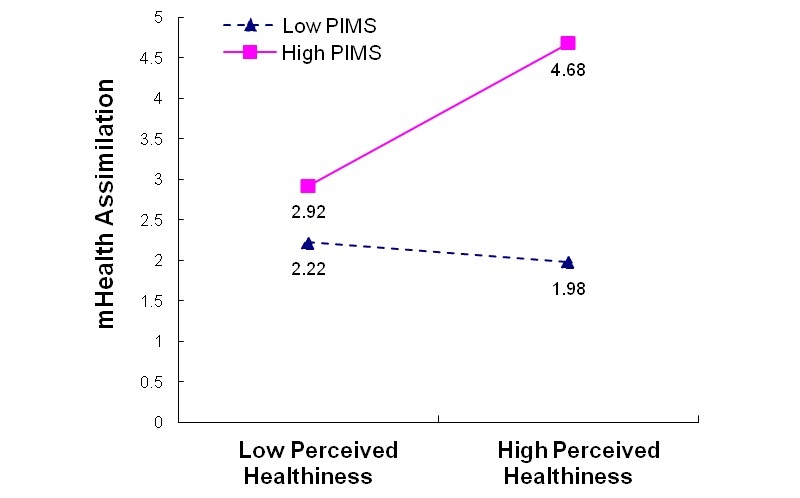
Moderating effect of personal innovativeness toward mobile services (PIMS) on perceived healthiness for mHealth usage assimilation: Model B4 PIMS*HLTH.

**Figure 3:**
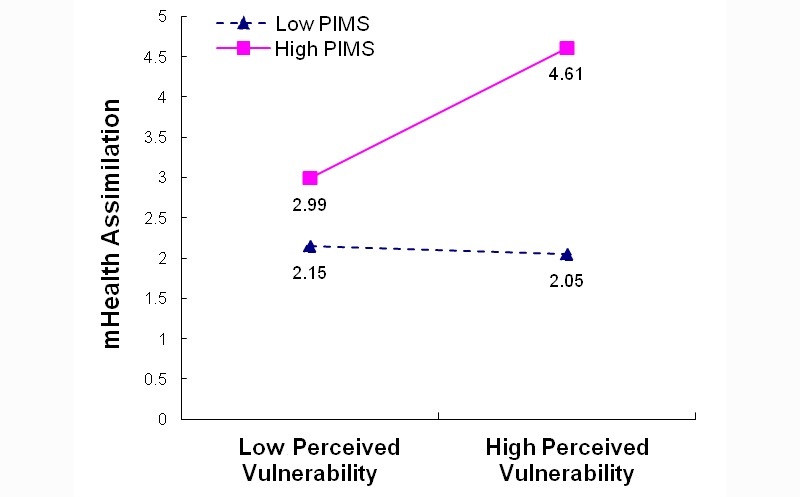
Moderating effect of personal innovativeness toward mobile services (PIMS) on perceived vulnerability for mHealth usage assimilation: Model B4 PIMS*VULN.

To develop a more nuanced understanding of the significant interaction effects in the mHealth assimilation model (B4), we plotted the interaction effects between PIMS and perceived healthiness (PIMS*HLTH) as well as between PIMS and perceived chronic disease vulnerability (PIMS*VULN). We performed simple slope tests of the effects of HLTH and VULN on assimilation at different levels of the moderator (ie, PIMS) as recommended by Aiken and West [76]. We observed that (1) respondents with high PIMS reported higher levels of assimilation when they reported higher health perceptions or higher perceived health vulnerability, whereas (2) respondents with low PIMS reported lower levels of assimilation than those with high PIMS, with the reported assimilation being even lower if respondents perceived themselves to be healthier or more vulnerable to chronic disease (Figures 2 and 3).

**Table 2:** Hierarchical ordinary least squares (OLS) regressions for consumer mHealth behavioral usage intention.

Variables		mHealth behavioral usage intention, OLS estimation (robust SE)			
		Model A1: Demographics and SES	Model A2: Health variables	Model A3: Personal innovativeness	Model A4: Interaction effects
**Demographics**					
	Age (continuous in years)	–0.02 (0.00)^c^	–0.02 (0.00)^c^	0.00 (0.00)	0.00 (0.00)
	Gender (female=1)	0.11 (0.10)	0.06 (0.09)	–0.00 (0.08)	–0.01 (0.08)
**Socioeconomic status**					
	Education (5=Master’s degree+)	0.00 (0.04)	0.05 (0.04)	0.03 (0.03)	0.03 (0.03)
	Individual income (5≥US $100K)	0.12 (0.04)^b^	0.03 (0.04)	–0.06 (0.04)	–0.06 (0.04)
**Dummy**					
	Adopter (1)/nonadopter (0)	2.33 (0.10)^c^	1.97 (0.11)^c^	1.17 (0.11)^c^	1.14 (0.11)^c^
**Health care availability**					
	Distance to primary facility	—	–0.05 (0.06)	–0.02 (0.05)	–0.03 (0.05)
	Distance to specialized facility	—	0.09 (0.05)	0.08 (0.05)	0.08 (0.05)
**Health care utilization**					
	Recent health checkup	—	0.04 (0.05)	–0.01 (0.04)	–0.01 (0.04)
**Perceived health conditions**					
	Perceived healthiness (HLTH)	—	0.30 (0.05)^c^	0.12 (0.05)^b^	0.10 (0.05)^a^
	Perceived vulnerability (VULN)	—	0.36 (0.05)^c^	0.18 (0.04)^c^	0.16 (0.05)^b^
**Personal innovativeness toward mobile services (PIMS)**					
	PIMS	—	—	1.11 (0.06)^c^	1.11 (0.06)^c^
	PIMS*HLTH	—	—	—	0.03 (0.04)
	PIMS*VULN	—	—	—	0.06 (0.04)
Constant		3.95 (0.21)^c^	4.05 (0.24)^c^	3.53 (0.20)^c^	3.50 (0.21)^c^
*R^2^*		0.4031	0.4406	0.5961	0.5970
∆*R^2^*		—	0.0375	0.1555	0.0009
*F*_df_ statistic		—	16.84_5,1121_^c^	358.13_1,1120_^c^	1.74_2,1118_

^a^ *P*<.05.

^b^ *P*<.01.

^c^ *P*<.001.

**Table 3:** Hierarchical ordinary least squares (OLS) regressions for consumer mHealth assimilation.

Variables		mHealth assimilation, OLS estimation (robust SE)			
		Model B1: Demographics and SES	Model B2: Health variables	Model B3: Personal innovativeness	Model B4: Interaction effects
**Demographics**					
	Age (continuous in years)	–0.04 (0.00)^c^	–0.04 (0.00)^c^	–0.02 (0.00)^c^	–0.01 (0.00)^c^
	Gender (female=1)	0.04 (0.12)	–0.10 (0.10)	–0.15 (0.09)	–0.16 (0.09)
**Socioeconomic status**					
	Education (5=Master’s degree+)	–0.11 (0.04)^a^	0.02 (0.04)	0.00 (0.04)	0.03 (0.03)
	Individual Income (5≥US $100K)	0.71 (0.06)^c^	0.38 (0.05)^c^	0.28 (0.05)^c^	0.24 (0.04)^c^
**Health care availability**					
	Distance to primary facility	—	0.09 (0.06)	0.11 (0.06)	0.05 (0.05)
	Distance to specialized facility	—	0.01 (0.05)	0.01 (0.05)	0.01 (0.04)
**Health care utilization**					
	Recent health checkup	—	0.17 (0.05)^b^	0.10 (0.05)^a^	0.10 (0.04)*
**Perceived health conditions**					
	Perceived healthiness (HLTH)	—	0.71 (0.06)^c^	0.51 (0.06)^c^	0.38 (0.06)^c^
	Perceived vulnerability (VULN)	—	0.90 (0.05)^c^	0.69 (0.05)^c^	0.43 (0.05)^c^
**Personal innovativeness toward mobile services (PIMS)**					
	PIMS	—	—	0.84 (0.57)^c^	0.85 (0.05)^c^
	PIMS*HLTH	—	—	—	0.50 (0.05)^c^
	PIMS*VULN	—	—	—	0.43 (0.04)^c^
Constant		4.09 (0.26)^c^	4.11 (0.25)^c^	3.27 (0.24)^c^	2.95 (0.23)^c^
*R* ^*2*^		0.2304	0.4486	0.5333	0.6041
∆*R* ^*2*^		—	0.2182	0.0847	0.0708
*F*_df_ statistic		—	71.15_5,1122_^c^	219.25_1,1121_^c^	151.24_2,1119_^c^

^a^ *P*<.05.

^b^ *P*<.01.

^c^ *P*<.001.

### mHealth Substitutive and Complementary Use Preference Results

Table 4 (models C1-C4) reports substitutive use preference regression results, and Table 5 (models D1-D4) reports complementary use preference regression results. In models C1 and D1, 27.48% and 18.43% of the variance was explained, respectively. Age was negatively associated with both substitutive use preference (beta=–0.03, *P*<.001) and complementary use preference (beta=–0.02, *P*<.001). In addition, individuals with higher levels of income were associated with higher substitutive use preference (beta=0.13, *P*=.003), but not complementary use preference (beta=0.05, *P*=.23). Moreover, females were more favorable to using mHealth as a complement to in-person doctor visits (beta=0.24, *P*=.009) than males, yet there was not a significant gender difference for using mHealth as a substitute to in-person doctor visits (beta=0.15, *P*=.11). The adopter group (adopter=1, nonadopter=0) was associated with higher substitutive use preference than nonadopters in all substitutive models (C1-C4) and in all complementary models (D1-D3), except for the interaction effects model (D4).

In models C2 and D2, health care access, health care utilization, and perceived health condition variables were added, increasing the explained variance (∆*R* ^*2*^) by 6.91% for substitutive use preference and by 4.52% for complementary use preference. Perceived healthiness and perceived vulnerability were positively related to both substitutive use preference (perceived healthiness: beta=0.30, *P*<.001; perceived vulnerability: beta=0.46, *P*<.001) and complementary use preference (perceived healthiness: beta=0.20, *P*<.001; perceived vulnerability: beta=0.34, *P*<.001). Although a recent health checkup was not significantly associated with complementary use preference (beta=0.03, *P*=.56), it was significantly negatively associated with substitutive use preference (beta=–0.16, *P*<.001).

In models C3 and D3, PIMS was included, increasing explained variance (∆*R* ^*2*^) by 9.56% for substitutive use preference and by 10.79% for complementary use preference. The positive and significant PIMS coefficients indicate that PIMS was a predictor of both substitutive use preference (beta=0.76, *P*<.001) and complementary use preference (beta=0.75, *P*<.001).

In models C4 and D4, the interaction between PIMS and perceived healthiness (ie, PIMS*HLTH) and the interaction between PIMS and perceived vulnerability (ie, PIMS*VULN) were added to the models, resulting in modest increases in explained variance (∆*R* ^*2*^) by 1.35% for substitutive use preference and by 0.55% for complementary use preference. Both interaction terms were significant predictors for complementary use preference (PIMS*HLTH: beta=0.19, *P*<.001; PIMS*VULN: beta=0.14, *P*<.001), whereas only PIMS*HLTH was a significant predictor for substitutive use preference (PIMS*HLTH: beta=0.12, *P*=.02 PIMS*VULN: beta=0.07, *P*=.11). Overall, these results indicate that PIMS and perceived health conditions jointly influence consumers’ preferences between in-person doctor visits and mHealth.

**Figure 4:**
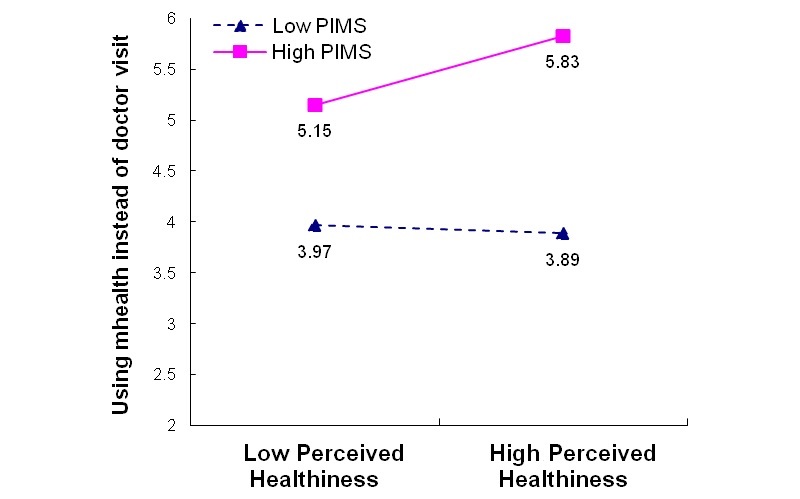
Moderating effect of personal innovativeness toward mobile services (PIMS) on perceived healthiness for preferring mHealth as a substitute to doctor visits: Model C4 PIMS*HLTH.

**Figure 5:**
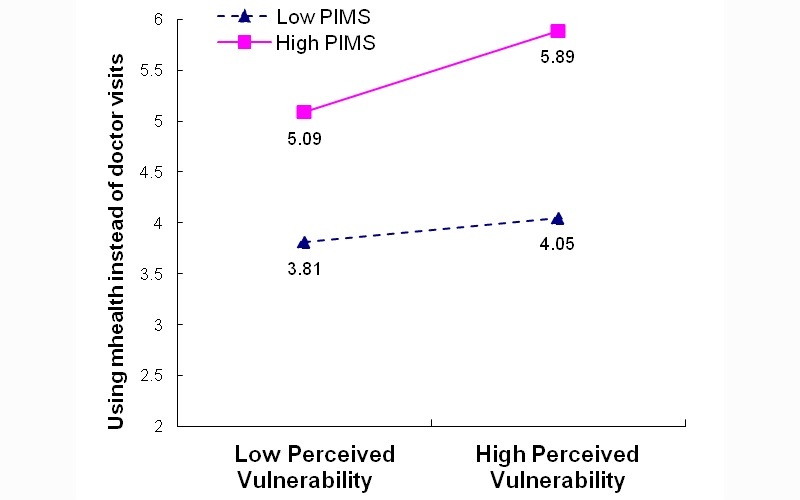
Moderating effect of personal innovativeness toward mobile services (PIMS) on perceived vulnerability for preferring mHealth as a substitute to doctor visits: Model C4 PIMS*VULN.

We again plotted the interaction effects and conducted simple slope tests. For respondents with high PIMS, we observed a greater preference for mHealth as a substitute to in-person doctor visits when they felt healthier or more vulnerable to chronic disease (Figures 4 and 5). In contrast, for respondents with low PIMS, those who felt healthier were marginally less likely to use mHealth as a substitute for in-person doctor visits, but those who felt more vulnerable were more likely to prefer mHealth as a substitute.

**Figure 6:**
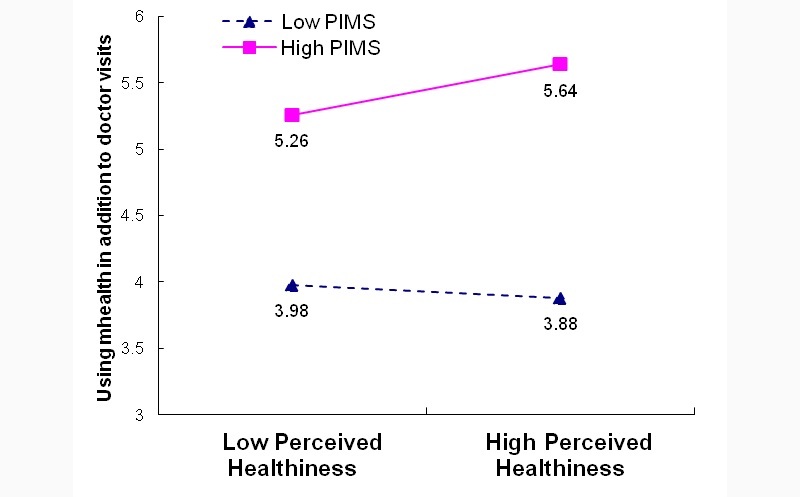
Moderating effect of personal innovativeness toward mobile services (PIMS) on perceived healthiness for preferring mHealth as a complement to doctor visits: Model D4 PIMS*HLTH.

As for the interaction effects associated with complementary use preference (Figure 6), respondents with high PIMS who felt healthier showed a greater preference for mHealth as a complement to in-person doctor visits relative to those who felt less healthy. For respondents with low PIMS, those who felt healthier indicated a marginally weaker preference for using mHealth as a complement relative to those who felt less healthy. The interaction between PIMS and VULN (PIMS*VULN) was not significant for model D4; thus, it is not presented here as a graph.

Given that the correlation of complementary use and substitutive use is 0.73, the preferences for complementary and substitutive use of mHealth can be interpreted to be mutually reinforcing. To further explore differences in those with stronger preferences for complementary use of mHealth than substitutive use of mHealth, we conducted an ANOVA analysis of differences in behavioral usage intention and assimilation between the following 2 groups: (1) stronger preference for complementary use than substitutive use (complementary > substitutive), and (2) stronger preference for substitutive use than complementary use (substitutive > complementary). We found that behavioral usage intention was significantly higher for the substitutive > complementary group (behavioral usage intention mean 4.69, SD 1.68) than for the complementary > substitutive group (behavioral usage intention mean 3.92, SD 1.79; *F*_1,702_=20.14, *P*<.001). Similarly, assimilation was significantly higher for the substitutive > complementary group (assimilation mean 3.78, SD 2.21) than for the complementary > substitutive group (assimilation mean 2.66, SD 1.66; *F*_1,702_=41.866, *P*<.001). Perceived health and perceived vulnerability to chronic disease were not significantly different between the 2 groups. These results reveal that although more respondents in our sample were willing to use mHealth as a complement than as a substitute for in-person doctor visits, the substitutive > complementary group indicated stronger behavioral usage intention and assimilation than the complementary > substitutive group. Additionally, respondents in the substitutive > complementary group, relative to the complementary> substitutive group, were younger (mean age 39.00 years, SD 14.20 vs 46.35 years, SD 16.81; *F*_1,702_=21.284, *P*<.001), had higher PIMS (PIMS mean 4.72, SD 1.68 vs 3.96, SD 1.76; *F*_1,702_=19.699, *P*<.001), and were less likely to be female (46%, SD 0.50 vs 55%, SD 0.50; *F*_1,702_=4.033, *P*=.045).

**Table 4:** Hierarchical ordinary least squares (OLS) regressions for mHealth substitutive use preference.

Variables		mHealth substitutive use preference, OLS estimation (robust SE)			
		Model C1: Demographics and SES	Model C2: Health variables	Model C3: Personal innovativeness	Model C4: Interaction effects
**Demographics**					
	Age (continuous in years)	–0.03 (0.00)^c^	–0.03 (0.00)^c^	–0.01 (0.00)^b^	–0.01 (0.00)^b^
	Gender (female=1)	0.15 (0.09)	0.14 (0.09)	0.10 (0.08)	0.09 (0.08)
**Socioeconomic status**					
	Education (5=Master’s degree+)	–0.05 (0.04)	0.01 (0.03)	–0.00 (0.03)	0.00 (0.03)
	Individual Income (5≥US $100K)	0.13 (0.04)^b^	0.06 (0.04)	–0.00 (0.04)	–0.01 (0.04)
**Dummy**					
	Adopter (1)/nonadopter(0)	1.42 (0.11)^c^	1.06 (0.11)^c^	0.50 (0.11)^c^	0.40 (0.11)^c^
**Health care availability**					
	Distance to primary facility	—	0.01 (0.06)	0.03 (0.05)	0.01 (0.05)
	Distance to specialized facility	—	0.03 (0.05)	0.02 (0.04)	0.02 (0.04)
**Health care utilization**					
	Recent health checkup	—	–0.16 (0.04)^c^	–0.20 (0.04)^c^	–0.19 (0.04)^c^
**Perceived health conditions**					
	Perceived healthiness (HLTH)	—	0.30 (0.05)^c^	0.18 (0.05)^c^	0.15 (0.05)^b^
	Perceived vulnerability (VULN)	—	0.46 (0.05)^c^	0.34 (0.05)^c^	0.26 (0.05)^c^
**Personal innovativeness toward mobile services (PIMS)**					
	PIMS	—	—	0.76 (0.06)^c^	0.78 (0.06)^c^
	PIMS*HLTH	—	—	—	0.19 (0.05)^c^
	PIMS*VULN	—	—	—	0.14 (0.04)^c^
Constant		4.72 (0.21)^c^	5.15 (0.22)^c^	4.79 (0.21)^c^	4.71 (0.21)^c^
*R* ^*2*^		0.2748	0.3439	0.4395	0.4530
∆*R* ^*2*^		—	0.0691	0.0956	0.0135
*F*_df_ statistic		—	24.91_5,1121_^c^	171.53_1,1120_^c^	14.12_2,1118_^b^

^a^ *P*<.05.

^b^ *P*<.01.

^c^ *P*<.001.

**Table 5:** Hierarchical ordinary least squares (OLS) regressions for mHealth complementary use preference.

Variables		mHealth complementary use preference, OLS estimation (robust SE)			
		Model D1: Demographics and SES	Model D2: Health variables	Model D3: Personal innovativeness	Model D4: Interaction effects
**Demographics**					
	Age (continuous in years)	–0.02 (0.00)^c^	–0.02 (0.00)^c^	–0.01 (0.00)^a^	–0.01 (0.00)^a^
	Gender (female=1)	0.24 (0.09)^b^	0.20 (0.09)^a^	0.16 (0.08)	0.15 (0.08)
**Socioeconomic status**					
	Education (5=Master’s degree+)	0.03 (0.03)	0.08 (0.03)^a^	0.07 (0.03)^a^	0.07 (0.03)^a^
	Individual income (5≥US $100K)	0.05 (0.04)	–0.02 (0.04)	–0.07 (0.04)	–0.08 (0.04)^a^
**Dummy**					
	Adopter (1)/nonadopter (0)	1.07 (0.09)^c^	0.76 (0.10)^c^	0.21 (0.10)^a^	0.15 (0.10)
**Health care availability**					
	Distance to primary facility	—	0.03 (0.06)	0.05 (0.05)	0.03 (0.05)
	Distance to specialized facility	—	0.05 (0.05)	0.05 (0.04)	0.05 (0.04)^a^
**Health care utilization**					
	Recent health checkup	—	0.03 (0.04)	–0.01 (0.04)	–0.01 (0.04)
**Perceived health conditions**					
	Perceived healthiness (HLTH)	—	0.20 (0.05)^c^	0.09 (0.05)	0.07 (0.05)
	Perceived vulnerability (VULN)	—	0.34 (0.05)^c^	0.22 (0.04)^c^	0.17 (0.05)^b^
**Personal innovativeness toward mobile services (PIMS)**					
	PIMS	—	—	0.75 (0.06)^c^	0.76 (0.06)^c^
	PIMS*HLTH	—	—	—	0.12 (0.05)^a^
	PIMS*VULN	—	—	—	0.07 (0.05)
Constant		5.09 (0.19)^c^	5.09 (0.22)^c^	4.73 (0.21)^c^	4.69 (0.21)^c^
*R* ^*2*^		0.1843	0.2295	0.3374	0.3429
∆*R* ^*2*^		—	0.0452	0.1079	0.0055
*F*_df_ statistic		—	15.43_5,1121_^c^	140.90_1,1120_^c^	4.00_2,1118_^a^

^a^ *P*<.05.

^b^ *P*<.01.

^c^ *P*<.001.

## Discussion

### Principal Findings and Implications

The market for mHealth is growing rapidly, but research in this emerging area has been limited. Before this study, there was limited understanding as to what determinants were associated with mHealth usage intentions, extent of mHealth assimilation, and why mHealth might be preferred as a complementary or substitutive service consumption channel in a context traditionally associated with hands-on, in-person interactions. This study has provided novel insights by examining how consumer usage intentions and assimilation of mHealth, as well as consumer channel preferences for health services are affected by PIMS, perceived health conditions, health care availability, health care utilization, demographics, and socioeconomic status.

Our primary findings are as follows: (1) more consumers are favorable to using mHealth as a complement to in-person doctor visits than as a substitute, but those who prefer mHealth as a substitute report stronger usage intentions and higher assimilation than those who prefer mHealth as a complement although not being significantly different in health perceptions, and (2) PIMS and perceived health conditions have direct effects on usage intentions, assimilation, and channel preferences and mutually reinforcing effects on assimilation and channel preferences. Of particular interest is the finding that the combination of higher PIMS and increased perceptions of healthiness as well as the combination of higher PIMS and increased perceptions of chronic disease vulnerability are significantly associated with higher mHealth assimilation and substitutive use of mHealth. The combination of higher PIMS and increased perceptions of healthiness is also significantly positively associated with the complementary use of mHealth. These interesting findings suggest that current health status is not the only predictor of mHealth usage and, perhaps counterintuitively, it is not necessarily those who perceive themselves as the least healthy who are the most likely to adopt and use mHealth.

We also demonstrate that PIMS and perceived chronic disease vulnerability are important positive predictors. Given these results, individuals worried about diet, weight, blood pressure, exercise, and other health issues might consider proactively using apps such as MyFitnessPal [77], InstantHeartRate [78], Macaw [79], and Livestrong [14]. Such proactive management of one’s health could significantly reduce the incidence of chronic disease and reduce the burden of such conditions on our health system [80].

Additionally, our results demonstrate that more than one-third of respondents specified current use of mHealth, whereas almost one-fifth of respondents report currently using mHealth on a regular basis. In addition, approximately two-thirds of respondents said they would use mHealth as a complement to in-person doctor visits. These findings elaborate prior research suggesting that individual innovativeness [50], individual traits [32], and health self-perceptions [81] are associated with usage intentions. Our findings extend prior research by considering the influence of these constructs, including the interaction effects of perceived health conditions, on consumers’ intentions, assimilation, and channel preferences for mHealth use. However, as suggested by the IT-enabled self-service literature, the infusion of technology into a service encounter may be met with resistance by those who prefer a hands-on relationship (vs a high-tech relationship) [46]. Our results support such findings in the mHealth context, in that health care utilization (ie, recent health checkup) had a positive effect on mHealth assimilation, but a negative effect on using mHealth as a substitute to in-person doctor visits. These results may indicate that personal relationships in health care settings will be difficult to augment (or replace) with technology for certain consumer segments. Although other technologies, such as telemedicine, have provided mechanisms to extend health services to those with limited access (eg, [82]), the issue of how mHealth can improve health care access while not adversely affecting patient-provider relationships will be an essential consideration.

Finally, we find that increased age is associated with decreased usage intentions and assimilation of mHealth in many of our models, whereas increased income is associated with increased usage intentions and usage of mHealth in some models. Similar findings have been reported in other technology acceptance studies [33,64,65]. Additionally, somewhat contrary to prior research suggesting that higher levels of education are often positively associated with technology adoption [65,66], we find a mix of significant and nonsignificant effects of education in our models. We did find that education was a positive and significant predictor within many of our models associated with using mHealth as a complement to in-person doctor’s office visits. This could be an area for further research.

### Strengths and Limitations

The strengths of our study include: (1) a theoretically driven model, based on technology acceptance, technology assimilation, consumer behavior, and health informatics literature, on the determinants of consumer mHealth usage intentions, assimilation, and channel preferences, (2) the inclusion of direct and interactive (moderating) effects of PIMS and perceived health conditions (vulnerability and healthiness) as determinants, and (3) robust survey, sampling, and analysis methods. Our study is limited by the cross-sectional nature of our survey. We note that our robustness checks included 2-stage estimation models and mediated models. All our findings held up to these checks, but future research could consider longitudinal research designs to elaborate our understanding of the mechanisms through which usage intentions and assimilation of mHealth develop. We are also limited by the use of an online survey, which may be biased toward those who complete surveys online or respondents who are more technologically sophisticated. Future research could consider other surveying and sampling strategies. Although our models have the feature of parsimony, they may exclude other situational, demographic, or individual characteristics. Future research could expand upon our findings by including such additional characteristics. Finally, our results are generalizable to the general population because the chosen sampling strategy and the use of statistical controls. However, future research could delve deeper into subgroup differences (adopters vs nonadopters, health respondents vs unhealthy respondents, resource-rich respondents vs resource-poor respondents, etc) and provide more nuanced findings regarding between and within group heterogeneity.

### Conclusions

This study has provided insights into the usage intentions, assimilation, and channel preferences associated with mHealth. These findings contribute to the health informatics literature and to health policy initiatives by demonstrating that mHealth will face both acceptance and resistance. Targeting the most receptive consumer segments may be the best strategy to encourage widespread diffusion. Multiple predictors have been shown to have significant impacts on mHealth preferences and both direct and interactive effects were observed. We suggest that future initiatives to promote mHealth should shift targeting of consumers from coarse demographics to individual dispositions toward mobile service innovations, complementary or substitutive channel use preferences, and perceived health conditions.

## Multimedia Appendix 1

Constructs and measurement sources.

## Multimedia Appendix 2

Reliability, average variance extracted, and correlations.

## Abbreviations

AVE: average variance extracted
CFA: confirmatory factor analysis
CMP: compatibility
HLTH: perceived healthiness
IT: information technology
OLS: ordinary least squares
PIIT: personal innovativeness toward information technology
PIMS: personal innovativeness toward mobile services
SES: socioeconomic status
VULN: perceived vulnerability

## References

[1] Lester RT, van der Kop M, Taylor D, Alasaly K, Coleman J, Marra F (2011). m-Health: Connecting patients to improve population and public health. BCMJ.

[2] Fox S, Duggan M (2012). Mobile health.

[3] PriceWaterhouseCoopers (2012). Emerging mHealth: Paths for Growth.

[4] Free C, Knight R, Robertson S, Whittaker R, Edwards P, Zhou W, Rodgers A, Cairns J, Kenward MG, Roberts I (2011). Smoking cessation support delivered via mobile phone text messaging (txt2stop): a single-blind, randomised trial. Lancet.

[5] Eysenbach G (2000). Consumer health informatics. BMJ.

[6] (2004). Center for Health Market Innovations.

[7] (2005). Center for Health Market Innovations.

[8] (2003). Center for Health Market Innovations.

[9] WebMD (2013). iTunes.

[10] SigmaPhone LLC (2012). iTunes.

[11] Mayo Clinic (2013). iTunes.

[12] (2013). eClinicalWorks.

[13] sanofi-aventis US LLC (2013). iTunes.

[14] Livestrong.com (2013). iTunes.

[15] WellDoc, Inc (2013). iTunes.

[16] Azumio Inc (2013). iTunes.

[17] Cocosila M, Archer N (2009). An empirical investigation of mobile health adoption in preventive interventions. BLED 2009 PROCEEDINGS.

[18] Hung MC, Jen WY (2012). The adoption of mobile health management services: an empirical study. J Med Syst.

[19] Weiner JP (2012). Doctor-patient communication in the e-health era. Isr J Health Policy Res.

[20] Cafazzo JA, Casselman M, Hamming N, Katzman DK, Palmert MR (2012). Design of an mHealth app for the self-management of adolescent type 1 diabetes: a pilot study. J Med Internet Res.

[21] Ohno-Machado L (2012). Informatics 2.0: implications of social media, mobile health, and patient-reported outcomes for healthcare and individual privacy. J Am Med Inform Assoc.

[22] Patrick K, Griswold WG, Raab F, Intille SS (2008). Health and the mobile phone. Am J Prev Med.

[23] Winstead-Derlega C, Rafaly M, Delgado S, Freeman J, Cutitta K, Miles T, Ingersoll K, Dillingham R (2012). A pilot study of delivering peer health messages in an HIV clinic via mobile media. Telemed J E Health.

[24] Baron J, Newman S (2011). A systematic review of the effectiveness of mobile health interventions for the management of diabetes.

[25] Schweitzer J, Synowiec C (2012). The economics of eHealth and mHealth. J Health Commun.

[26] Curioso WH, Mechael PN (2010). Enhancing 'M-health' with south-to-south collaborations. Health Aff (Millwood).

[27] Kahn JG, Yang JS, Kahn JS (2010). 'Mobile' health needs and opportunities in developing countries. Health Aff (Millwood).

[28] Demiris G (2012). New era for the consumer health informatics research agenda. Health Syst.

[29] Whittaker R (2012). Issues in mHealth: findings from key informant interviews. J Med Internet Res.

[30] Hsieh J, Rai A, Keil M (2008). Understanding digital inequality: Comparing continued use behavioral models of the socio-economically advantaged and disadvantaged. MIS quarterly.

[31] Agarwal R, Prasad J (1998). A conceptual and operational definition of personal innovativeness in the domain of information technology. Information Systems Research.

[32] Agarwal R, Prasad J (1999). Are individual differences germane to the acceptance of new information technologies?. Decision Sciences.

[33] Or CK, Karsh BT (2009). A systematic review of patient acceptance of consumer health information technology. J Am Med Inform Assoc.

[34] Thompson TG, Brailer DJ (2004). The Decade of Health Information Technology: Delivering Consumer-Centric and Information-Rich Health Care.

[35] Davis FD, Bagozzi RP, Warshaw PR (1989). User acceptance of computer technology: a comparison of two theoretical models. Management Science.

[36] Fichman RG, Kemerer CF (1997). The assimilation of software process innovations: an organizational learning perspective. Management Science.

[37] Rai A, Brown P, Tang X (2009). Organizational assimilation of electronic procurement innovations. Journal of Management Information Systems.

[38] Dholakia UM, Kahn BE, Reeves R, Rindfleisch A, Stewart D, Taylor E (2010). Consumer behavior in a multichannel, multimedia retailing environment. Journal of Interactive Marketing.

[39] Nysveen H (2005). Mobilizing the brand: the effects of mobile services on brand relationships and main channel use. Journal of Service Research.

[40] Becker M (1974). The health belief model and sick role behavior. Health Educ Monogr.

[41] Bethell C, Fiorillo J, Lansky D, Hendryx M, Knickman J (2004). Online consumer surveys as a methodology for assessing the quality of the United States health care system. J Med Internet Res.

[42] Janz NK, Becker MH (1984). The Health Belief Model: a decade later. Health Educ Q.

[43] Venkatesh V, Thong J, Xu X (2012). Consumer acceptance and use of information technology: extending the unified theory of acceptance and use of technology. MIS quarterly.

[44] Fichman RG, Zmud RW (2000). The diffusion assimilation of information technology innovations. Framing the Domains of IT Management: Projecting the Future...Through the Past.

[45] Berry LL, Seiders K, Grewal D (2002). Understanding service convenience. Journal of Marketing.

[46] Bitner MJ, Brown SW, Meuter ML (2000). Technology infusion in service encounters. Journal of the Academy of Marketing Science.

[47] Keh HT, Pang J (2010). Customer reactions to service separation. Journal of Marketing.

[48] Agarwal R, Prasad J (1998). The antecedents and consequents of user perceptions in information technology adoption. Decision Support Systems.

[49] Klein R (2007). An empirical examination of patient-physician portal acceptance. Eur J Inf Syst.

[50] Lu J, Yao JE, Yu C (2005). Personal innovativeness, social influences and adoption of wireless Internet services via mobile technology. The Journal of Strategic Information Systems.

[51] Thatcher JB, Perrewe PL (2002). An empirical examination of individual traits as antecedents to computer anxiety and computer self-efficacy. MIS Quarterly.

[52] Agarwal R, Sambamurthy V, Stair RM (2000). Research report: the evolving relationship between general and specific computer self-efficacy--an empirical assessment. Information Systems Research.

[53] Goldsmith RE (2002). Explaining and predicting consumer intention to purchase over the internet: an exploratory study. Journal of Marketing Theory and Practice.

[54] Goldsmith RE (2001). Using the Domain Specific Innovativeness Scale to identify innovative Internet consumers. Internet Research.

[55] Citrin AV, Sprott DE, Silverman SN, Stem DE, Jr (2000). Adoption of Internet shopping: the role of consumer innovativeness. Industrial Management & Data Systems.

[56] Kuo Y, Yen S (2009). Towards an understanding of the behavioral intention to use 3G mobile value-added services. Computers in Human Behavior.

[57] Lu J, Yao JE, Yu C (2005). Personal innovativeness, social influences and adoption of wireless Internet services via mobile technology. The Journal of Strategic Information Systems.

[58] Moore GC, Benbasat I (1991). Development of an instrument to measure the perceptions of adopting an information technology innovation. Information Systems Research.

[59] Plouffe CR, Vandenbosch M, Hulland J (2001). Intermediating technologies and multi-group adoption: A comparison of consumer and merchant adoption intentions toward a new electronic payment system. Journal of Product Innovation Management.

[60] Rosenstock IM (2005). Why people use health services. Milbank Quarterly.

[61] Bloch PH (1995). Seeking the ideal form: product design and consumer response. Journal of Marketing.

[62] Emont S (2011). Measuring the impact of patient portals: what the literature tells us.

[63] Agarwal R, Anderson C, Zarate J, Ward C (2013). If we offer it, will they accept? Factors affecting patient use intentions of personal health records and secure messaging. J Med Internet Res.

[64] Bigne E, Ruiz C, Sanz S (2005). The impact of internet user shopping patterns and demographics on consumer mobile buying behaviour. Journal of Electronic Commerce Research.

[65] Li H, Kuo C, Russell M (1999). The impact of perceived channel utilities, shopping orientations, and demographics on the consumer's online buying behavior. J Comput Mediat Commun.

[66] Liao Z, Cheung MT (2001). Internet-based e-shopping and consumer attitudes: an empirical study. Information & Management.

[67] Déglise C, Suggs LS, Odermatt P (2012). Short message service (SMS) applications for disease prevention in developing countries. J Med Internet Res.

[68] Källander K, Tibenderana JK, Akpogheneta OJ, Strachan DL, Hill Z, ten Asbroek AH, Conteh L, Kirkwood BR, Meek SR (2013). Mobile health (mHealth) approaches and lessons for increased performance and retention of community health workers in low- and middle-income countries: a review. J Med Internet Res.

[69] (2012). United States Census Bureau.

[70] Lindell MK, Whitney DJ (2001). Accounting for common method variance in cross-sectional research designs. J Appl Psychol.

[71] Anderson JC, Gerbing DW (1988). Structural equation modeling in practice: A review and recommended two-step approach. Psychological Bulletin.

[72] Hair JF (1995). Multivariate Data Analysis with Readings.

[73] Segars A (1997). Assessing the unidimensionality of measurement: a paradigm and illustration within the context of information systems research. Omega.

[74] Fornell C, Larcker DF (1981). Evaluating structural equation models with unobservable variables and measurement error. Journal of Marketing Research.

[75] Nunnally J, Bernstein IH (1994). Psychometric Theory.

[76] Aiken LS, West SG, Reno RR (1991). Multiple Regression: Testing and Interpreting Interactions.

[77] MyFitnessPal.com (2013). iTunes.

[78] Azumio Inc (2013). iTunes.

[79] US Preventive Medicine, Inc (2013). iTunes.

[80] Bodenheimer T, Lorig K, Holman H, Grumbach K (2002). Patient self-management of chronic disease in primary care. JAMA.

[81] Houston TK, Allison JJ (2002). Users of Internet health information: differences by health status. J Med Internet Res.

[82] Boulanger B, Kearney P, Ochoa J, Tsuei B, Sands F (2001). Telemedicine: a solution to the followup of rural trauma patients?. J Am Coll Surg.

