# Supplementary material for: Understanding Determinants of Consumer Mobile Health Usage Intentions, Assimilation, and Channel Preferences
Source: J Med Internet Res. 2013 Aug 2;15(8):e149. doi: 10.2196/jmir.2635 (PMC3742412; doi:10.2196/jmir.2635)
Supplement: Supplementary file 1 [file jmir_v15i8e149_app1.pdf]

Understanding Determinants of Consumer Mobile Health (mHealth) Usage Intentions, Assimilation, and Channel Preferences

MULTIMEDIA APPDENIX 1: Constructs and Measurement Sources

Constructs	Items	Item Scale	Sources
Behavioral Intention to use mHealth (BI)	Assuming I have access to mobile services related to healthcare, I intend to use them.	1-Strongly Disagree to 7-Strongly Agree	[1, 2]
	If I have access to mobile services related to healthcare, I predict that I would use them.	1-Strongly Disagree to 7-Strongly Agree	
mHealth Assimilation (ASSIM)	Please characterize your level of use of mobile services for healthcare services.	Not aware; aware but no plan to use; aware but plan to use in the near future; less than once per month; a few times per month; weekly, daily, multiple times per day	[3]
Substitutive (SUB) Willingness-to-Use mHealth	I am willing to use mHealth services for obtaining health advice instead of in-person doctor visits.	1-Strongly Disagree to 7-Strongly Agree	Self-developed (based on concepts from [4])
	I am willing to use mHealth services for monitoring compliance for treatment instead of in-person doctor.	1-Strongly Disagree to 7-Strongly Agree	
	I am willing to use mHealth services for exchanging clinical information with healthcare providers instead of in-person doctor visits.	1-Strongly Disagree to 7-Strongly Agree	
Complementary (COMP) Willingness-to-Use mHealth	I am willing to use mHealth services for obtaining health advice in addition to in-person doctor visits.	1-Strongly Disagree to 7-Strongly Agree	Self-developed (based on concepts from [4])
	I am willing to use mHealth services for monitoring compliance for treatment in addition to in-person doctor visits.	1-Strongly Disagree to 7-Strongly Agree	
	I am willing to use mHealth services for exchanging clinical information with healthcare providers in addition to in-person doctor visits.	1-Strongly Disagree to 7-Strongly Agree	
Personal Innovativeness toward Mobile Services (PIMS)	If I heard about a new mobile service, I would look for ways to experiment with it.	1-Strongly Disagree to 7-Strongly Agree	[5]
	Among my friends and family, I am usually the first to try out new mobile services.	1-Strongly Disagree to 7-Strongly Agree	
	I like to experiment with new mobile services.	1-Strongly Disagree to 7-Strongly Agree	

Perceived Healthiness (HLTH)	I feel I am (very unhealthy/ very healthy).	1-Very Unhealthy to 7-Very Healthy	Self-developed (based on concepts from the health belief model [6])
Perceived Health Vulnerability (VULN)	I feel vulnerable to severe chronic diseases (i.e., Diabetes/Heart Disease/ Cancer/ High Blood Pressure/ Stroke) in the next five years.	1-Strongly Disagree to 7-Strongly Agree	Self-developed (based on concepts from the health belief model [6])
Recency of Health Checkup (CHEK)	Have you had a health checkup?	No; yes, within the past five years; yes, within the past three years; yes, within the past one year	Self-developed
Distance to primary healthcare facilities	Please indicate how close the primary healthcare facilities are from your home.	Less than 1 mile; 1-5 miles; 6-10 miles; greater than 11 miles; do not know	Self-developed
Distance to specialized healthcare facilities	Please indicate how close the specialized healthcare facilities are from your home.	Less than 1 mile; 1-5 miles; 6-10 miles; greater than 11 miles; do not know	Self-developed

References

1. Agarwal R, Prasad J. Are individual differences germane to the acceptance of new information technologies? Decision Sciences 1999;30(2):361-91. [doi:10.1111/j.1540-5915.1999.tb01614.x]

2. Venkatesh V, Davis F. A theoretical extension of the technology acceptance model: Four longitudinal field studies. Management Science 2000;46(2):186-204. [doi:10.1287/mnsc.46.2.186.11926]

3. Davis FD. User Acceptance of Information Technology: System Characteristics, User Perceptions and Behavioral Impacts. International Journal of Man-Machine Studies 1993;38(3):475-87. [doi:10.1006/imms.1993.1022]

4. Nysveen H, Pedersen PE, Thorbjørnsen H, Berthon P. Mobilizing the Brand The Effects of Mobile Services on Brand Relationships and Main Channel Use. Journal of Service Research 2005;7(3):257-76. [doi:10.1177/1094670504271151]

5. Agarwal R, Prasad J. A Conceptual and Operational Definition of Personal Innovativeness in the Domain of Information Technology. Information Systems Research 1998;9(2):204-15. [doi:10.1287/isre.9.2.204]

6. Janz N, Champion V, Strecher V. The health belief model. In: Glanz K, Rimer B, editors. Health behavior and health education. San Francisco: Jossey-Bass 2002.
