# Supplementary material for: Understanding Determinants of Consumer Mobile Health Usage Intentions, Assimilation, and Channel Preferences
Source: J Med Internet Res. 2013 Aug 2;15(8):e149. doi: 10.2196/jmir.2635 (PMC3742412; doi:10.2196/jmir.2635)
Supplement: Supplementary file 2 [file jmir_v15i8e149_app2.pdf]

Understanding Determinants of Consumer Mobile Health (mHealth) Usage Intentions, Assimilation, and Channel Preferences

MULTIMEDIA APPENDIX 2: Reliability, Average Variance Extracted, and Correlations

	Constructs	Mean	Std.	α [a]	C.R. [b]	AVE [c]	1	2	3	4	5	6	7	8	9	10	11	12	13
1	BI [d]	4.09	2.02	.98	.87	.78	.88[e]												
2	ASSIM [f]	3.20	2.23	-	-	-	.65	-											
3	SUB	4.17	1.77	.92	.92	.80	.61	.54	.89										
4	COMP	4.87	1.63	.95	.95	.87	.57	.42	.73	.93									
5	PIMS	4.18	1.93	.94	.94	.84	.73	.62	.61	.55	.92								
6	HLTH [f]	5.29	1.46	-	-	-	.30	.37	.26	.20	.33	-							
7	VULN [f]	4.01	2.02	-	-	-	.24	.34	.26	.21	.18	-.19	-						
8	CHEK [f]	2.49	0.99	-	-	-	.09	.13	-.04	.05	.07	.08	.13	-					
9	AGE [f]	45.44	16.20	-	-	-	-.34	-.34	-.36	-.30	-.49	-.19	.09	.16	-				
10	GEN [f]	-	-	-	-	-	-.06	-.11	-.04	.01	-.07	-.05	.02	.10	.13	-			
11	EDU [f]	-	-	-	-	-	.08	.09	.03	.07	.09	.09	-.11	.03	-.01	-.12	-		
12	INC [f]	-	-	-	-	-	.22	.35	.18	.13	.26	.29	.07	.13	-.05	-.23	.41	-	
13	PRIM [f]	-	-	-	-	-	.03	.09	.02	.05	-.03	-.03	.21	.13	.16	.02	-.01	.06	-
14	SPEC [f]	-	-	-	-	-	.04	.06	.00	.04	-.03	.03	.13	.19	.19	.00	.05	.08	.59

Notes:

[a] Cronbach's Alpha

[b] Composite Reliability

[c] Average Variance Extracted

[d] BI: Intention to use mHealth (multi-item measure); ASSIM: mHealth assimilation (single-item measure); SUB: Substitutive use of mHealth (multi-item measure); COMP: Complementary use of mHealth (multi-item measure); PIMS: Personal innovativeness toward mobile services (multi-item measure); HLTH: Perceived healthiness (single-item measure); VULN: Perceived vulnerability (single-item measure); CHEK: Recency of health checkup (single-item measure); AGE: Age (years); GEN: Gender (M=0, F=1); EDU: Education (1 to 5, 1= Primary School; 5=Master's degree or higher.); INC: Individual income (1 to 5; 1=Less than $24,999; 5=$100,000 or more); PRIM: Distance to primary facility (miles); SPEC: Distance to specialized facility (miles).

[e] Diagonals represent the square root of the average variance extracted. The off-diagonal elements are inter-construct correlations.

[f] Single item, categorical variable (therefore, means, standard deviations, average variances extracted, and reliabilities are not reported)
